# Processing sites in the human immunodeficiency virus type 1 (HIV-1) Gag-Pro-Pol precursor are cleaved by the viral protease at different rates

**DOI:** 10.1186/1742-4690-2-66

**Published:** 2005-11-01

**Authors:** Steve C Pettit, Jeffrey N Lindquist, Andrew H Kaplan, Ronald Swanstrom

**Affiliations:** 1 Department of Medicine, University of North Carolina at Chapel Hill, Chapel Hill, NC, USA; 2 Department of Biochemistry and Biophysics, University of North Carolina at Chapel Hill, Chapel Hill, NC, USA; 3 The UNC Center for AIDS Research, University of North Carolina at Chapel Hill, Chapel Hill, NC, USA; 4 CB7295, Rm 22-006 Lineberger Bldg, UNC Center For AIDS Research, University of North Carolina at Chapel Hill, Chapel Hill, NC 27599-7295, USA; 5 Department of Pathology, Moores UCSD Cancer Center, 3855 Health Sciences Dr. #0803, La Jolla, CA 92093-0803, USA; 6 3805-103 Chimney Ridge Pl., Durham, NC, 27713, USA

## Abstract

We have examined the kinetics of processing of the HIV-1 Gag-Pro-Pol precursor in an in vitro assay with mature protease added in trans. The processing sites were cleaved at different rates to produce distinct intermediates. The initial cleavage occurred at the p2/NC site. Intermediate cleavages occurred at similar rates at the MA/CA and RT/IN sites, and to a lesser extent at sites upstream of RT. Late cleavages occurred at the sites flanking the protease (PR) domain, suggesting sequestering of these sites. We observed paired intermediates indicative of half- cleavage of RT/RH site, suggesting that the RT domain in Gag-Pro-Pol was in a dimeric form under these assay conditions. These results clarify our understanding of the processing kinetics of the Gag-Pro-Pol precursor and suggest regulated cleavage. Our results further suggest that early dimerization of the PR and RT domains may serve as a regulatory element to influence the kinetics of processing within the Pol domain.

## Findings

The retroviral protease (PR) processes the Gag and Gag-Pro-Pol precursors during the assembly of the mature virus particle. The viral structural proteins assume altered conformations after processing, and the viral enzymes become fully active in their processed forms [1-7]. Proper proteolytic processing is necessary for assembly of an infectious particle [3,4,8-10].

Cleavage of Gag is ordered and appears to be regulated, at least in part, by the target site sequence, the presence of spacer domains, and the interaction with RNA [8,9,11,12]. Previous studies showed the five HIV-1 Gag processing sites are cleaved at rates that vary up to 400-fold in vitro [9,13]. Initial cleavage occurs at the p2/NC site followed by an intermediate rate of cleavage at the MA/CA and p1/p6 sites, and final cleavage at the CA/p2 and NC/p1 sites [9,12-16]. A similar pattern of ordered processing appears to occur in infected cells [9,12,17,18].

**Figure 1:**
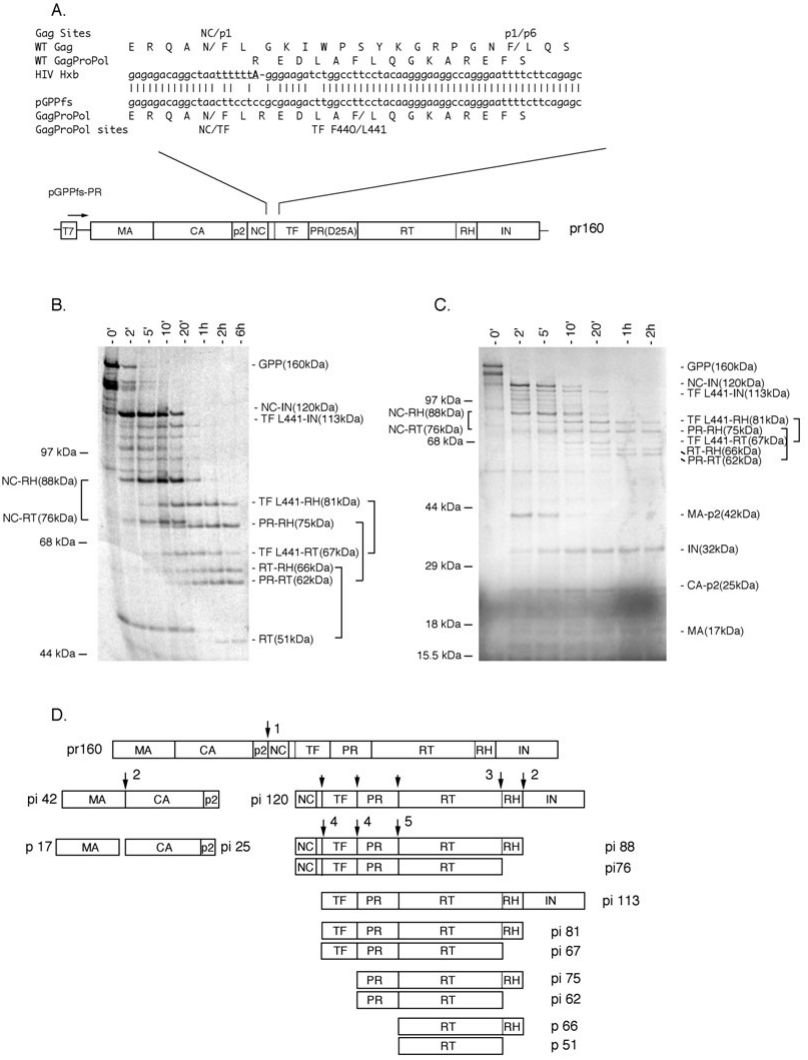
A. The frameshift mutation in pGPPfs-PR. Above: the sequence of wild type HIV-1 HXB (GenBank:NC001802) molecular clone in the area of translational frameshift in *gag*-*pro*-*pol *is shown. The heptanucleotide slippery sequence required for translational frameshifting is underlined [23, 24]. The adenine that is read twice during frameshifting is shown in bold. The exact site of frameshifting in the wild type virus is variable with 70% of Gag-Pro-Pol product containing Leu as the second residue of the transframe domain (TF) [27]. pGPPfs-PR expressed in vitro in a coupled transcription/translation system [28] gives the predominant Gag-Pro-Pol product. Additional translationally silent substitutions were inserted in the area frameshift to reduce secondary structure and translational pausing during expression. The activity of the intrinsic protease was inactivated by a D25A substitution of the catalytic aspartate. The location of the Gag NC/p1 [53] and pl/p6 [54] sites and the Gag-Pro-Pol NC/TF and TF F440/L441 sites [28, 32, 33, 35] are also shown. Below: an overall schematic pGPPfs-PR. B, C. Processing of the HIV-1 Gag-Pro-Pol precursor in vitro showing the kinetics of processing and the generation of product pairs over time. The full-length Gag-Pro-Pol pr160 precursor containing an inactive protease (by PR D25A mutation of the catalytic aspartate) was generated by transcription and translation of pGPPfs-PR in a rabbit reticulocyte lysate. Purified mature HIV-1 protease was added in trans following the 0' timepoint. Aliquots were removed at the indicated time and the protein products separated by Tris-Glycine SDS-PAGE (B) [30] or by Tris-Tricine SDS-PAGE (C) [31]. Paired products resulting from prior removal of IN followed by partial cleavage at the RT/RH site are denoted with brackets. Molecular mass markers are shown on the left. The molecular masses of the intermediates and final products, as estimated from published sequence or common nomenclature, are also shown. Products are represented in abbreviated form by the N- and C-terminal domains according to the nomenclature of Leis et al. [55]. D. Proposed pathway for the ordered processing of the HIV-1 Gag-Pro-Pol precursor by protease in trans. The Gag-Pro-Pol precursor and the observed predominant processing intermediates are represented as boxes with processing sites denoted as vertical lines. The schematic separates the observed Gag-Pro-Pol cleavages into distinct rates. The initial cleavage at p2/NC is shown with a large arrow and labeled 1. The next cleavages occur with similar rates and are labeled 2 (RH/IN and MA/CA). This cleavage is quickly followed by half-cleavage at the RT/RH site (labeled 3). A series of intermediates between 120 kDa and 88 kDa are accounted for at least in part by early cleavage at the sites upstream of RT (TF F440/L441, TF/PR, PR/RT), and these are indicated with small arrows. The slower cleavages at these sites (labeled 4 and 5) give rise to the later paired products. The molecular masses shown of the intermediates and final products were estimated from published sequence or common nomenclature.

Processing of the HIV-1 Gag-Pro-Pol precursor by protease in trans is less studied, although the final cleavage products [MA, CA, NC, transframe (TF), PR, RT, IN] are well characterized [19-22]. The HIV-1 Gag-Pro-Pol precursor results from a -1 frameshift event during translation at a site near the 3' end of the *gag *reading frame to join the *gag *and *pro-pol *reading frames [23,24]. For this study, we created by site-directed mutagenesis [25,26] a continuous HIV-1 *gag-pro-pol *reading frame that would produce a full-length precursor identical in sequence to the viral Gag-Pro-Pol polyprotein precursor [23,27] (Fig. 1A). Intrinsic protease activity was inactivated by a D25A substitution of the catalytic aspartate of the PR domain to produce the final construct GPPfs-PR (Fig. 1A). We expressed the radio-labeled Gag-Pro-Pol using an in vitro transcription/translation strategy [9,28] and monitored cleavage at known processing sites as a function of time after adding 0.25 μg recombinant HIV-1 protease (as described in [13,28,29]) in a reaction volume of 50 μl. Under these conditions the concentration of precursor is approximately 0.1 nM. Products were separated using two different SDS-PAGE systems [30,31] prior to autoradiography.

Fig. 1B and 1C show the pattern of cleavage products generated at different time points after the addition of protease in trans. We identified over ten distinct species greater than 50 kDa (Fig 1B). Fig. 1C shows products of lower molecular mass [31]. The combination of two different gel systems allowed for the separation and analysis of the appearance of each product. An initial species of 120 kDa (processing intermediate pi120) was rapidly generated within 2 minutes then disappeared to form distinct intermediates of 88, 81, 76, 75, 67, 62 kDa, and finally the mature RT products p66 and p51 (Fig. 1B, C). We observed a large difference in the rates of appearance of these intermediates. After 6 hours of incubation six processing intermediates remained even though the first cleavage event to generate pi120 occurred within 2 min (Fig 1B), indicating that the sites are cleaved at highly different rates. No observable processing occurred without added protease (data not shown), indicating that processing was due to the added protease. Thus, processing of the Gag-Pro-Pol precursor results in a processing cascade consisting of discrete intermediates.

We have used three strategies to assign the cleavage sites that define the ends of the processing products. The first we assigned the products based on the known processing sites in Gag-Pro-Pol. The size of the pi120 intermediate was consistent with an initial cleavage at the p2/NC site, the same site initially cleaved in the Gag precursor [9,14-16]. Second, we truncated the Gag-Pro-Pol precursor to establish the polarity of the initial cleavage site. We implicated cleavage at the p2/NC site by truncating 116 residues from the C-terminal end of the precursor via linearization of the template by Afl II prior to RNA synthesis in vitro. Protease cleavage of the truncated precursor resulted in a shift of the pi120 intermediate to 110 kDa (data not shown), a size consistent with initial cleavage at the p2/NC site. Third, in order to confirm the site of cleavage and the identification of products we blocked individually blocked cleavage at the p2/NC, TF/PR, PR/RT, RT/RH and RH/IN sites by site-directed mutagenesis as described (data not shown) [9,13]. Each blocking mutation resulted in alternative unprocessed intermediates with a molecular mass consistent with an absence of cleavage at the mutated site. Thus, this approach supported the identification of the cleavage sites and the intermediates presented here. We noted that each site was generally cleaved independently of the other sites by protease in trans. A notable exception was the CA/p2 site which showed enhanced cleavage when the earlier cleaved p2/NC site was blocked (M377I mutation). Previously, we reported similar enhanced cleavage of this site in the Gag precursor with the same blocking mutation at the p2/NC site [9]. There is a series of faint minor products between pi120 and pi88, at 113 kDa, 107 kDa, 100 kDa, and 95 kDa (Fig. 2A) seen at the 2-minute time point. These likely represent a low level of cleavage at all of the known cleavage sites upstream of RT early in the processing cascade. We showed by mutagenesis that 113 kDa intermediate resulted from cleavage at the TF F440/L441 site (Fig. 1A, and 1D) rather than cleavage at the NC/TF (data not shown). The TF F440/L441 site has previously been identified as a processing site by others [32-34] using less than full length Pol precursors, and this site is cleaved by the activated PR within full length Gag-Pro-Pol [17,28,35,36]. Other intermediates in this group are likely accounted for as PR-IN (107 kDa) and RT-IN (97 kDa) products.

We observed four sets of paired intermediates and products (denoted by brackets in Fig. 1B, C). We interpret these pairs to represent intermediates that resulted from full cleavage at the RH/IN site followed by half cleavage at the RT/RH site. Numerous studies have shown that partial cleavage of the RT/RH site in the purified RT-RH homodimer is dependent on the dimerization of the RT domain to induce unfolding of a single RH domain [19,21,22,37-40]. We observed a similar pattern with the full length Gag-Pro-Pol precursor, with IN removed prior to half cleavage of the RT/RH cleavage site, also in agreement with [41] where an *E. coli *based expression system was used. Thus, by analogy with the results of others, we infer that the RT domain within the expressed Gag-Pro-Pol precursor is dimeric either prior to or immediately after removal of IN. The pi88/pi76 paired products, derived from pi120, appeared initially at the 2 minute time point showing that RH/IN and RT/RH cleavage occur relatively early in the processing cascade. The later and overlapping appearance of the three remaining product pairs showed that subsequent N-terminal processing of the pi88/pi76 pair is ordered, but occurs at more similar rates. The SDS-PAGE system utilized in Fig. 1B allows for separation of the pi76 and pi75 intermediates and shows the disappearance of the pi88/pi76 paired products follows the 20 minute time point. The pi81/pi67 and pi75/pi62 pairs represent later products that likely result from cleavage at the TF F440/L441 and TF/PR sites, respectively. Lastly, the mature p66/p51 products represent final cleavage at the PR/RT site.

Initial cleavage at the p2/NC site also generated a MA-CA-p2 (pi42) product (Fig. 1C). We previously showed that cleavage of p42 in vitro occurs at the MA/CA cleavage site followed by slower cleavage at the CA/p2 site [9,13]. We observe here that the rates of processing of the MA/CA and RH/IN sites are similar as shown by the similar appearance of pi25 CA-p2 and p32 IN (Fig. 1C).

Fig. 1D summarizes a proposed cascade for processing of Gag-Pro-Pol by mature protease in trans. The initial cleavage occurs at the p2/NC site (presumably at the same rate this site is cleaved in Gag), generating the pi120 NC-TF-PR-RT-RH-IN intermediate and the p42 MA-CA-p2 intermediate. The next cleavage removes IN from the C terminus of pi120 by cleavage at RH/IN producing pi88. Removal of IN occurs at a rate similar to cleavage between MA-CA. Cleavage of RH/IN is closely followed by cleavage of the RT/RH site to generate the initial paired pi88 and pi76 NC-TF-PR-RT (RH) products. The presence of these paired products suggests that dimerization of the RT-containing processing intermediate occurred early in the processing cascade, consistent with the results of others who observed a similar cleavage pattern using more fully processed dimeric RT [22,38,40]. Processing at the TF F440/L441 and TF/PR occur next followed by the final cleavage between PR/RT to generate the final mature PR and RT products. Final cleavage of the precursor occurs in the sites flanking the PR domain, suggesting that accessibility to these sites may be restricted via formation of a dimer interface structure similar to that observed in mature protease [42].

The overall pattern and extent of processing differs substantially with protease present in trans compared to the pattern seen with the protease embedded in the precursor, as previously characterized [28,35,36]. Cleavage of the Gag-Pro-Pol precursor by the embedded protease appears to be much more restrictive with cleavages only observed at the p2/NC site and the TF F440/L441 sites. We show here that protease present in trans cleaves all of the Gag-Pro-Pol sites but at varying rates (Figs. 1B, C, D), resulting in a processing cascade. One possibility is that the embedded protease shows restricted site selection due to its location within the precursor.

We infer that the Gag-Pro-Pol precursor was able to dimerize in this expression system. The state of the Gag-Pro-Pol precursor in newly assembled (or assembling) virions could differ. In infected cells, Gag-Pro-Pol may dimerize while moving to the assembly site [43-46] or during assembly, affecting the kinetics of precursor processing. Alternatively, dimerization of Gag-Pro-Pol monomer may be constrained by the excess of Gag during assembly, as suggested by others [47-49]. In that case, the presence of Gag could limit Gag-Pro-Pol dimerization by forming heterodimers, in turn altering the kinetic of processing. These considerations are not mutually exclusive. One of the early cleavage events detailed here (such as cleavage at p2/NC) could also release a truncated precursor from a Gag/Gag-Pro-Pol heterodimer and permit rapid dimerization of the PR and RT domains.

The other feature of the system we have used is the reliance of protease cleavages in trans. Use of trans protease on the full length precursor allows for the clear evaluation of generation of each product, however, this approach is unable to discern the possible cleavage of nascent or truncated products or the effect of an active embedded protease. Expression of Gag-Pro-Pol in vitro with an unmutated protease domain results in rapid autocatalytic cleavage at the p2/NC site and the TF F440/L441 site to produce the 113 KDa intermediate [28,35]. Immediate dimerization in cells of the full length precursor would likely result in premature cleavage [50-52]. Thus, in the context of budding virions there may be an interplay between monomeric versus dimeric Gag-Pro-Pol as substrate, and embedded versus free protease for cleavage. The extent to which these different combinations may alter the order of cleavage and the successful assembly of virus is not known.

We show here that cleavage of the Gag-Pro-Pol processing sites by trans protease occurs at different rates, and we suggest that cleavage is likely regulated, in part, by the dimerization of the protease and RT domains. We and others have shown that timed and ordered cleavage of the HIV-1 Gag precursors is highly regulated and is necessary for the production of an infectious, properly assembled virion. We do not yet know the extent of the requirement for timed cleavage of Gag-Pro-Pol in producing infectious virus. Characterization of the ordered cleavage of Gag-Pro-Pol furthers our understanding of HIV-1 precursor processing and suggests further mechanisms at work in the regulation of HIV-1 assembly.

### Competing interests

The author(s) declare that they have no competing interests.

## Authors' contributions

JL and SP carried out the experiments. RS and SP drafted the manuscripts and designed the experiments. AK provided helpful discussion and editing of the manuscript.
